# Bioactive Plant Peptides: Physicochemical Features, Structure-Function Insights and Mechanism of Action

**DOI:** 10.3390/molecules30183683

**Published:** 2025-09-10

**Authors:** Sara Avilés-Gaxiola, Israel García-Aguiar, Luis Alfonso Jiménez-Ortega, Erick Paul Gutiérrez-Grijalva, José Basilio Heredia

**Affiliations:** 1 Health Sciences Department, Universidad Autónoma de Occidente, Blvd. Lola Beltrán and Blvd. Rolando Arjona, Culiacán 80020, Mexico; sara.aviles@uadeo.mx (S.A.-G.); israel.garcia@uadeo.mx (I.G.-A.); 2 Centro de Investigación en Alimentación y Desarrollo, AC Nutraceutical and Functional Foods, Carretera a Eldorado Km 5.5, Col. Campo el Diez, Culiacán 80110, Mexico; ljimenez222@estudiantes.ciad.mx; 3 Cátedras CONACYT—Centro de Investigación y Desarrollo, AC Carretera a Eldorado Km 5.5, Col. Campo el Diez, Culiacán 80110, Mexico; erick.gutierrez@ciad.mx

**Keywords:** plant bioactive peptides, structure, function, diseases, health

## Abstract

Different cultures worldwide have attributed particular healing abilities to various plants for a long time. After decades of studies, research has demonstrated that their bioactivity is associated mainly with the presence of natural products, including short protein fragments known as peptides. These molecules may occur naturally in plants or be generated from plant protein through enzyme hydrolysis. In recent years, a growing body of evidence has linked plant-derived peptides to diverse biological activities, underscoring the importance of their structural and physicochemical features in determining functionality. Compared with peptides of animal or microbial origin, plant peptides stand out for their high abundance in sustainable sources, low allergenic potential, and distinctive structural traits- such as enrichment in hydrophobic and aromatic residues- that influence their stability, mechanisms of action, and biological functions. This review compiles and analyzes current literature to provide insights into how amino acid composition, secondary structure, net charge, and hydrophobicity influence peptide bioactivity. In addition, the review highlights the mechanisms of action most frequently described for plant peptides. Finally, the article discusses the current landscape and prospects of peptide-based drugs.

## 1. Introduction

For a long time, popular culture has established that certain foods and plants help alleviate various chronic ailments. However, in recent years, the role of specific dietary compounds in preventing diseases and improving human quality of life has become evident. In recent decades, the search for bioactive molecules has intensified, linking their activity with specific mechanisms of action [1]. Peptides are one of the most reported natural compounds with nutraceutical potential. They are linear chains composed of fewer than 40 amino acids joined by covalent bonds and released from a more complex protein structure called the parent protein [2]. Due to their size, peptides may be absorbed in the intestine, accessing the bloodstream, allowing these molecules to exert a physiological effect in vivo. They have been found to impact body functions and influence health positively, boosting the quality of human health, and therefore have been considered bioactive molecules, i.e., nutraceuticals [3]. These molecules are sometimes called cryptides, bioactive peptide fragments encrypted within larger protein sequences and released through enzymatic hydrolysis [4]. Furthermore, peptides have minimal side effects since they are not accumulated in organs and are absent from immunoreactions [5]. Over 5300 bioactive peptides have been cataloged in the BIOPEP-UWM^tm^ database [6].

Protein-rich foods are considered optimal sources for the extraction of peptides. Within this category, animal-derived foods are particularly prominent, encompassing milk, eggs, cheese, meat, fish industry by-products, and even bovine blood [7]. In recent years, bioactive peptide research has focused on alternative sources, such as those derived from protein-rich plants. This shift is mainly because producing plant-based proteins is more economical and sustainable than animal sources. Additionally, plants are plentiful, and the demand for plant-derived products continues to rise, as their consumption is linked to healthier dietary choices [8]. Moreover, agri-food byproducts have emerged as promising reservoirs of bioactive peptides, offering an opportunity to valorize waste streams while supporting circular economy approaches [9]. Beyond these advantages, plant peptides display distinctive features compared to animal- or microbe-derived peptides. They are generally associated with lower allergenic potential, which enhances their safety in nutraceutical and functional food applications [10]. Structurally, they often show a higher prevalence of hydrophobic and aromatic residues—particularly at the C-terminal—that improve their stability, antioxidant potential, and interactions with molecular targets [11]. These differences, combined with their sustainability and accessibility, highlight plant peptides as a unique and promising class of bioactive molecules. In this context, plant peptides have been found to display anti-thrombotic, antimicrobial, antihypertensive, immunomodulatory, antioxidative, and anticancer activities, among many others [12].

Bioactive peptides are mainly produced from plant proteins through enzymatic hydrolysis, gastrointestinal digestion, and microbial fermentation [13,14,15]. Enzymatic hydrolysis of proteins is the most common and effective method for producing bioactive peptides. This process involves using either a single enzyme or a combination of different enzymes. The most frequently used proteinases are digestive enzymes, such as pepsin, trypsin, chymotrypsin, and pancreatic enzymes. Additionally, enzymes derived from fungal and bacterial sources, such as alcalase and plant-based enzymes like papain, are also utilized in this process [16]. Each enzyme has a particular active site within the protein; consequently, the degree of hydrolysis of a specific enzyme depends on controllable and optimizable factors such as hydrolysis time, enzyme concentration, and enzyme combination [17]. Once produced, peptides require purification and characterization. Ultrafiltration and chromatographic methods are the principal strategies for isolation, whereas advanced techniques such as liquid chromatography coupled with UV detection or tandem mass spectrometry (MS/MS) enable structural identification [13,14,15,16,17]. These technological improvements have greatly expanded the discovery of new bioactive peptides and insights regarding their structure-function [18,19,20]. Research has shown that the activity of peptides is closely related to their chemical structure and the conformation of their amino acids. This review seeks to analyze the structure–activity relationship of plant peptides based on our current knowledge and analyses. Understanding this relationship is essential for developing therapeutic peptides, which offer an innovative, effective, and promising alternative for treating prevalent diseases in today’s population.

## 2. Structure-Function Relationship of Bioactive Plant Peptides

### 2.1. Relation Between Plant Peptide Structure and Its Antioxidant Activity

Oxidative stress is an imbalance caused by a higher concentration of oxidants than antioxidants. This phenomenon breaks redox signaling, promoting cellular damage [21]. Reactive oxygen species (ROS), such as O_2_^−^, HO_2_, H_2_O_2_, and OH^−^, damage biomolecules, including DNA, proteins, and lipids; the damage caused by oxidative stress can both promote the onset of chronic diseases as well as their complications [22]. Over the past few decades, considerable interest has been focused on antioxidants, particularly those of natural origin [23]. Plant peptides are among the most extensively studied natural antioxidant molecules, emerging as a trending and prominent research area. Since 2010, 98 research articles have been published evaluating the antioxidant potential of plant peptides, with around 40 of these papers identifying the plant peptide sequences. Note that the primary methods used to determine the antioxidant activity of peptides are chemical-based, iron chelating activity, and radical scavenging (ABTS and DPPH). Cell-based methods have also been developed, such as cellular antioxidant activity assays using various cell lines like HepG2 and Caco-2 [24]. In Table 1, the antioxidant activity of plant peptides is presented. After our analysis, we determined that the peptides with higher antioxidant activity repeat key amino acids such as glutamic acid, aspartic acid, glycine, alanine, leucine, and phenylalanine. Glutamic acid and aspartic acid are negatively charged amino acids; since they have excess electrons, they have free radical quenching activity. Also, due to their charged residues, they chelate metals and inhibit metal-mediated oxidation [25,26]. On the other hand, these peptides show the most potent lipid peroxidation activity [27].

Glycine, alanine, and leucine are aliphatic amino acids that provide structural flexibility and contribute to peptide–lipid interactions, enhancing cellular uptake [11,75]. In contrast, antioxidant capacity is primarily attributed to residues with aromatic or sulfur-containing side chains—such as tyrosine, tryptophan, phenylalanine, cysteine, and methionine—which can donate electrons or hydrogen atoms to neutralize free radicals. Histidine, with its imidazole group, also plays a key role in radical scavenging. Importantly, the position of these within the peptide sequence is critical: antioxidant activity is enhanced when hydrophobic or aromatic amino acids are located at the C-or N-terminal nds, improving solubility in lipid systems and protecting membranes from peroxyl radical–mediated oxidation [76,77].

For example, the hydrophobic amino acid leucine reduces Fe_3_^+^, mainly when located at the N and C-terminal, increasing DPPH radical scavenging activity. Leucine also contributes to antioxidant activity through its long aliphatic side chain, which interacts with the acyl chains of fatty acids [56]. Regarding phenylalanine, it has been reported that this aromatic amino acid provides protons from its benzene group, acting directly as a radical scavenger and promoting high values on the assays ABTS and ORAC [78]. Tyrosine contains a phenolic group, which acts as a hydrogen atom donor, providing protons to electron-deficient free radicals and quenching them in *in vitro* methods such as the DPPH assay. Also, the most effective position for tyrosine has been reported in the C-terminal. This amino acid also scavenges the peroxyl radicals generated during the AAPH assay. It has also been associated with high CAA values since it can remove peroxyl radicals more easily than other amino acids [29]. It has been reported that peptides containing tyrosine have twice the antioxidant activity as those without it in their structure [62].

The sulfur-containing amino acids, methionine and cysteine, have a nucleophilic character and have been found to contribute to the scavenging activity of peptides by sulfur-hydrogen donation [71]. Cysteine is another important amino acid since it directly interacts with free radicals because its thiol group protects cellular molecules from oxidation with high TEAC assay values [30,38,62]. Also, peptides containing cysteine possess higher ferric antioxidant power [57].

Nevertheless, since plant protein is deficient in sulfur-containing amino acids, they are not commonly found in all plant-derived peptides [41]. Another amino acid recognized for its antioxidant activity is histidine, a radical scavenger due to its imidazole group proton-donating ability [41]. Histidine’s contribution to peptides’ antioxidant activity is higher when located in the C-terminus position. Finally, valine at the N-terminal has been related to enhancing the activity of antioxidant peptides in oil systems [36].

Besides particular amino acids interacting with free radicals, particular peptides may influence gene expression. One is SOD-3, a crucial endogenous antioxidant enzyme, which is the case for the peptide FDPAL obtained from soybean [69]. In addition, other soy peptides were able to activate the Keap1/Nrf2 pathway, also increasing the expression of antioxidant and phase II enzymes such as SOD1, TrxR1, NQO1, and GR [70].

A relationship between peptides having a secondary structure and their antioxidant activity has been established, being higher with a lower random coil content [51]. Also, lower α-helix content has been reported to be linked with increased antioxidant capacity [41]. As for the length, antioxidant plant peptides have been reported to have 2 to 21 amino acids. We found that up to 66% of the identified antioxidant plant peptides have from 2 to 10 amino acids. This is very convenient because smaller peptides can easily pass through cell membranes. Also, short peptides may pass through the intestinal barrier and exert their effect in the body. The reported peptides have been found not to cause damage to healthy cells; cytotoxic and hemolytic assays have seen this. Plant peptides should be utilized as medicinal antioxidants and as nutraceuticals for functional foods to increase antioxidant activities and prevent food oxidation reactions, increasing shelf life, for example, in high lipid foods. Antioxidant plant peptides are an alternative to synthetic antioxidants considered harmful, such as BHA, BHT, and TBHQ, which affect the spleen, lung, and liver [74].

### 2.2. Relation Between Plant Peptides Structure and Its Antiproliferative Activity

Cancer is a process involving an uncontrolled division of the body’s cells. It is one of the most relevant health problems globally, one of the leading causes of death. The short size of peptides allows them to penetrate cell membranes, where they can interact with oncogenic proteins inside the cell [79]. Also, they are efficacious signaling molecules that bind to specific cell surface receptors, such as ion channels. These actions trigger cell cycle arrest, suppress cancer cell growth, and inhibit invasion through various mechanisms, including cytoplasmic membrane disruption, mitochondrial membrane depolarization, DNA damage, and autophagy-like cell death [80,81].

Bowman-Birk trypsin inhibitor is an 8 kDa naturally occurring peptide isolated from various legumes and highlighted for its antiproliferative activity, which is attributed to its ability to inhibit serine proteases. This function is due to the presence of two inhibitory domains in its structure: CTKSNPPTC and CAYSNPPKC. The specific residues responsible for this activity are lysine (in the first domain) and tyrosine (in the second domain). Additionally, the high abundance of cysteine in this peptide facilitates the formation of disulfide bonds, enhancing its stability and resistance [82].

It has been established that the amino acids that repeat the most among plant peptides reported with anticancer activity are glutamic acid, leucine, serine, phenylalanine, and alanine (Table 2). As reported in the literature, associations between these amino acids and anticancer activity in peptides are detailed below. As for glutamic acid, it is a negatively charged amino acid and has been recognized to have antiproliferative activity on tumor cells [83,84]. Leucine and alanine are hydrophobic amino acids, improving peptide cell penetration [85]. Although the serine anticancer mechanism is not fully understood, Chatupheeraphat et al. [79] reported that removing serine from anticancer peptide sequences significantly reduced bioactivity. Peptides containing phenylalanine have a higher affinity for cancer cell membranes because phenylalanine contributes to the hydrophobicity of these peptides [83]. Hydrophobic and hydrophilic amino acids in the C or N-terminal promote an amphiphilic character, which has been reported to be beneficial. While hydrophobic amino acids are necessary to invade cancer cell membranes, hydrophilic ones give the peptide stability and a high possibility of interaction inside the cell [86]. Peptides containing hydrophobic amino acids (>30%) and also amino acids that give the peptide a positive charge (greater than or equal to 1), for example, lysine and arginine, bind to the membranes of cancer cells, which have a net negative charge associated with the presence of the phosphatidylserine phospholipid, which accounts for 9% of the total amount of phospholipids in the human cell membrane. Usually, this phospholipid faces the cytoplasm, but it goes to the outer part of the membrane in cancer cells, giving them a net negative charge. Once they attach to the membrane, peptides with these characteristics create pores through electrostatic interactions. This process leads to the leakage of intracellular components, resulting in necrosis. In the case of non-cationic peptides, the presence of hydrophobic amino acids enables them to penetrate the cell interior and interact with cytoplasmic regulatory proteins [79,87].

Other important peptide amino acids with anticancer activity include proline, histidine, tryptophan, and glycine. The presence of proline increases the flexibility of peptides [99]. Histidine enables peptides to induce cancer cytotoxicity by increasing membrane permeability. Tryptophan has been found to enter cancer cells by the endocytic pathway and bind to the major groove of nuclear DNA [83]. On the other hand, glycine-rich peptides have been found to stimulate NK cell activation, enhancing antitumor activity in both animal and human models, although the exact mechanism has not been established [106].

Amino acids described in the antioxidant section are also essential in the structure of anticancer peptides, as they prevent cells from being in an oxidative stress state and play a role in anti-tumor activity [97,107]. It has been previously reported that antioxidant peptides can potentially prevent and treat ROS-related diseases, particularly certain types of cancer. These peptides can act as anticancer compounds by inhibiting oxidative stress and reducing genetic alterations, such as mutations and chromosomal rearrangements [96]. As for the secondary structure, β-pleated sheet peptides usually have two or even more disulfide bonds and therefore are more stable than α-helical peptides [106]. As for the length of the identified anticancer plant peptides, they range from 3 to 158 amino acids.

Nevertheless, we have determined that approximately 63% of the identified anticancer plant peptides contain between 3 and 10 amino acids. Small peptides exhibit greater molecular mobility compared to those of longer length, and they have high diffusivity across membranes and a higher probability of interacting with cancer cell molecules within the cells. Peptides longer than 20 amino acids have been found to possess less cytotoxic effect on cancer cells. Molecular docking studies have shown that specific peptides interact with the active site of enzymes involved in regulating gene expression, such as HDAC1 and MDM2. In this context, the quinoa-derived peptide HYNPYFPG forms hydrogen bonds with the active site of HDAC1, thereby inhibiting the enzyme’s activity. Histidine and aspartic acid were identified as the key amino acids responsible for this inhibitory effect [96]. Regarding MDM2, the rapeseed-derived peptide NDGNQPL exhibited high affinity for the enzyme’s active site, promoting its inhibition primarily through establishing hydrophobic interactions [98].

Different mechanisms have been associated with plant peptides, such as the mitochondrial apoptotic pathway (rapeseed, sweet potato, pecan, soybean, and maize) and autophagic cell death (walnut peptides). On the other hand, bean peptides specifically affected gene expression in colon cancer cells HCT116 and RKO. They mainly upregulated transcriptionally activated genes that encode for antioxidant enzymes related to NRF-2, associated with cancer prevention. Also, it affected genes involved in MAPK signaling, having a role in proliferation and apoptosis [88]. Bean peptides also promote DNA damage through PARP cleavage and cell cycle arrest through nuclear translocation of p53 [81]. Another reported mechanism is for *Pombalia calceolaria* peptides, which inhibited cancer cells’ migration [95].

### 2.3. Relation Between Plant Peptides Structure and Its Angiotensin-Converting Enzyme Inhibitory Activity

The feasibility of using bioactive peptides as potential hypotensive drugs depends on their bioavailability and bioactivity, Plant bioactive peptides can act as hypotensive drugs by known mechanisms. However, one of the most studied is their capacity to inhibit the angiotensin-converting enzyme (ACE). The renin-angiotensin and the kinin-nitric oxide systems are the hypotensive activity mechanisms that have been studied the most. However, the most well-studied bioactive peptides are the renin-angiotensin system, where the angiotensin-converting enzyme is one of the main protagonists [108,109]. The angiotensin-converting-enzyme, a glycosylated membrane-bound zinc metalloprotease, is the main protagonist of the renin-angiotensin system; in normal conditions, its primary metabolic function begins after renin acts on angiotensinogen to angiotensin I, an inactive peptide which is later catalyzed by the ACE to angiotensin II and then binds to vascular wall receptors to cause the contraction of the blood vessels. However, during hypertensive conditions, the abnormal conditions cause the renin-angiotensin system to function excessively, leading to high levels of angiotensin II, thus causing hypertension [110,111]. Therefore, the angiotensin-converting enzyme inhibition can help lower the blood pressure in hypertensive patients [112,113]. Moreover, most recent studies have shown that bioinformatic predictions can help to model peptides with specific bioactivities like antihypertensive biopeptides [114].

Previous studies have elucidated a correlation between the amino acid sequences and their ACE-inhibitory capacity (Table 3) [109]. In another related subject, one mode of action of bioactive peptides in the ACE inhibition is competition of the peptides with the ACE substrate for the union with the enzyme’s catalytic site. This site contains three specific pockets, and the interaction between peptides and ACE involves various types of molecular forces, such as van der Waals interactions, hydrophobic contacts, and electrostatic attractions. Among these, hydrogen bonds are predominant in stabilizing the peptide–ACE complex [115]. According to studies employing molecular docking tools, amino acids such as isoleucine, leucine, and proline enhance the antihypertensive activity of peptides. This effect is attributed to their ability to establish specific interactions with residues like histidine, alanine, and arginine, respectively, which are located within the binding pockets of the ACE. These interactions hinder the enzyme’s catalytic activity, thereby contributing to its inhibition [116,117]. On the other hand, A net negative charge resulting from the incorporation of strongly acidic amino acids, such as aspartic acid and glutamic acid, can potentially interfere with ACE activity by binding to the zinc ion essential for its catalytic function [118].

**Figure 1:**
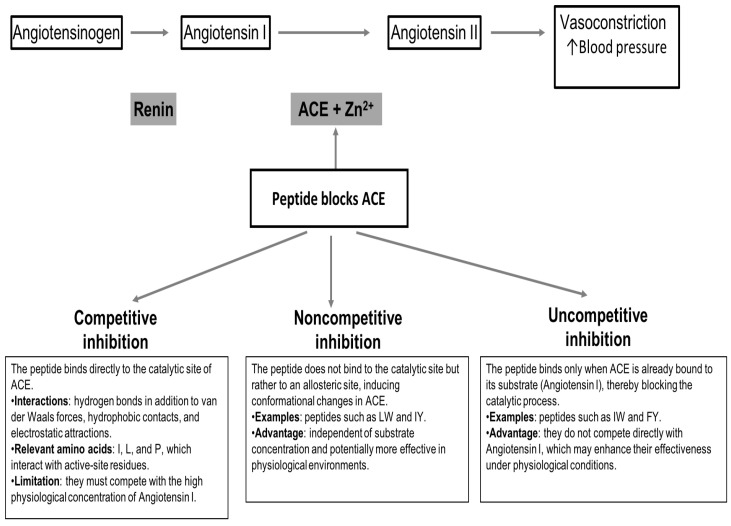
Schematic representation of the inhibitory mechanisms of plant-derived peptides on ACE.

The above is not always the case, for some bioactive peptides like LW and IY, a noncompetitive inhibition has been reported. In the case of the peptides IW and FY, they have been cataloged as uncompetitive inhibitors [109,118]. It is worth noting that competitive ACE inhibitors have a practical limitation, as they must compete with the high physiological concentration of the natural substrate, angiotensin I. In this regard, noncompetitive and uncompetitive peptides like those mentioned above, which do not rely on substrate competition, may offer greater effectiveness [111]. The different mechanisms by which plant-derived peptides inhibit ACE are illustrated in a schematic representation in Figure 1.

Hydrophobic and aliphatic amino acids are also necessary for peptides’ hypotensive activity [109]. Most ACE-inhibitory peptides are characterized by the presence of hydrophobic or branched-chain amino acids at the N-terminus. At the same time, residues with cyclic or aromatic rings—such as tyrosine, phenylalanine, tryptophan, and proline—are commonly found at the C-terminus. In fact, structural analyses of hypotensive peptides have shown that approximately 40% contain tryptophan at the C-terminal end [115]. One of the main reasons for the prevalence of hydrophobic amino acids in these bioactive peptides is the hydrophobic nature of the binding pockets in the ACE, which enhances interactions and binding affinity with peptides possessing such characteristics [112,117,118].

Peptide size also plays a critical role in determining bioactivity. In this regard, it has been documented that smaller peptides, particularly those composed of fewer than ten amino acids, exhibit a more potent antihypertensive effect compared to larger peptides. One contributing factor is that shorter peptides—especially dipeptides and tripeptides—can be absorbed intact through the gastrointestinal tract and reach the blood circulatory system. Additionally, specific amino acid residues, such as proline, phenylalanine at the C-terminal, and leucine at the N-terminal, have been shown to confer resistance to peptidase degradation. These residues also enhance affinity for peptide transporters, further facilitating absorption. Moreover, the smaller size of these peptides increases their likelihood of effectively interacting with the active site of ACE [121].

Moreover, it is essential to mention that unless there is sufficient and significant data regarding the pharmacological potential of bioactive peptides from plant origin, absorption, distribution, metabolism, and excretion studies are important. Unfortunately, this approach is often neglected, whether by instrumental or budget restrictions. Also, studies should focus on mimicking physiological conditions and concentrations of peptides, because the aleatory use of peptide concentrations in *in vitro* studies might lead to an overestimation of their antihypertensive potential [128,129]. Thus, an experimental design considering the abovementioned factors is needed for more physiologically relevant results.

### 2.4. Relation Between Plant Peptides Structure and Its Hypolipidemic Activity

Dyslipidemia is a metabolic disorder characterized by abnormal concentrations of lipids and lipoproteins in the bloodstream, most commonly involving elevated levels of total cholesterol, low-density lipoprotein cholesterol (LDL-C), triglycerides, or decreased levels of high-density lipoprotein cholesterol (HDL-C). It represents a significant risk factor for cardiovascular diseases and is influenced by genetic, dietary, and lifestyle factors, as well as enzymatic activity involved in lipid metabolism and various underlying pathological conditions.

Peptides isolated from soybean and lupine have been shown to affect 3-hydroxy-3-methylglutaryl coenzyme A reductase (HMGCoAR), since they act as competitive inhibitors of the enzyme. This effect is due to characteristics such as the number, position, and type of residues of amino acids, as well as peptide hydrophobicity. For example, proline and valine have been reported as crucial, and the inhibitory activity is higher when they are located within the first and the fourth N-terminal positions and when they are flanked by leucine, phenylalanine, alanine, and glycine [130,131].

By Molecular docking and *in vitro* studies, lupine and rapeseed peptides (Table 4) were found to inhibit HMGCoAR, low-density lipoprotein receptor (LDLR), and proprotein convertase subtilisin/kexin type-9 (PCSK9).

As for PCSK9 inhibition, it was reported that the amino acid leucine within the peptide LPKHSDAD (Table 4) was inserted in the hydrophobic pocket of PCSK9, forming a stable peptide-PCSK9 union stabilized by hydrogen bonds [142,145]. Plant peptides containing at least four hydrophobic amino acids have a hypocholesterolemic effect [151]. Mainly if one is located at the C- or N-terminal [152,153,154,155]. Hydrophobic amino acids establish hydrophobic interactions with lipids and with the non-polar molecules of bile acids, which, although weak, promote the reduction in blood cholesterol levels [131,156,157,158]. On the other hand, peptides with cationic amino acids can also capture bile acids because the cationic residues interact with the carboxyl groups of bile acids. Some plant peptide sequences have been modified to increase their hypocholesterolemic activity by adding polar amino acids like serine residues to the C-terminal, improving their solubility in aqueous systems [155,159]. We determined that the amino acids that repeat the most among peptides with hypolipidemic activity are lysine, threonine, valine, glutamic acid, and isoleucine. These peptides are mainly made up of 2 to 20 amino acids.

### 2.5. Relation Between Plant Peptides Structure and Its Hypoglycemic Activity

Diabetes is a degenerative pathology that causes chronic hyperglycemia due to apoptosis of the pancreatic β-cells. If it is not controlled, cardiovascular diseases and other health complications can develop [160,161]. Peptides with hypoglycemic activity are characterized by inhibiting carbohydrate metabolism enzymes such as α-amylase, α-glucosidase, and DPP-IV. In addition, they can also act by inhibiting the glucose transporter system and acting as insulin-like molecules [162,163]. Table 5 shows plant sources of hypoglycemic peptides.

Notably, in the inhibition of α-glucosidase, the most potent plant peptides correspond to those containing three to six amino acids [174]. Similarly, another relevant factor is the properties of the amino acid residues at the N-terminal and C-terminal. In this regard, the analysis of several articles has led to the conclusion that α-glucosidase inhibitory activity is higher when arginine, tyrosine, lysine, threonine, or serine is located at the final N-terminal residue and when methionine or alanine is situated at the final C-terminus [175]. Another outstanding characteristic that has been observed is the presence of proline. Some investigations have documented that proline and a basic amino acid, such as lysine and arginine, can increase the bioactivity against α-glucosidase.

Regarding their net charge, peptides with 0 or +1 are the most effective in inhibiting α-glucosidase [191,192,193]. Docking simulation has shown that a glycine-serine-arginine sequence inhibits α-glucosidase by attaching to the pocket of the enzyme due to van der Waals forces [175,189,190]. The enzymes with which the hydrolysis is carried out play an important role in defining the biological activity of the peptides. Plant hydrolysates obtained with alkaline protease show the highest α-glucosidase inhibition rate [126]. It is worth mentioning that the most studied peptide inhibitors of α-glucosidase are between 5 and 6 amino acids; the tetrapeptides are the most potent inhibitors of this enzyme [194].

Plant peptides containing proline or alanine residues inhibited DPP-IV enzyme through competitive inhibition for its active site [195,196,197]. Regarding DPP-IV inhibition, the amino acids alanine and leucine have the most potential to interact with the catalytic site through hydrogen bonds and polar and non-polar interactions [171,184]. Also, peptides with high inhibition of DPP-IV contain isoleucine in the N1 position. This may be because isoleucine favors the formation of α-helix [172].

On the other hand, amino acids such as serine, threonine, and tyrosine interact with the α-amylase active site due to their hydroxyl group, inhibiting it. Furthermore, higher α-amylase inhibitory activities have been reported when these amino acids are located at the N-terminal position. On the other hand, proline or alanine near or at the C-terminal position promotes the formation of hydrogen bonds and electrostatic interactions of plant peptides with the catalytic site of α-amylase [187]. Our literature research found that the reports associate the amino acids glycine, alanine, valine, threonine, and proline with the most abundant amino acids in peptides with hypoglycemic activity.

### 2.6. Relation Between Plant Peptide Structure and Its Antimicrobial Activity

Medicinal plants used in traditional medicine are attractive sources of bioactive proteins and peptides that demonstrate a broad spectrum of activities, including antimicrobial [198]. Usually, antimicrobial peptides (AMPs) are broad-spectrum agents that act on bacteria, fungi, metazoans, and other parasites [199]. Most AMPs are short protein fragments composed of 10 to 50 amino acids with a net positive charge from +2 to +11. It has been observed that 50% of AMPs contain hydrophobic residues, which allows them to have a strong affinity to net negatively charged microbial membranes [200].

The enormous variety of plant AMPs causes difficulty in their classification. Considering the secondary structures, AMPs are classified into “α” family (with α-helical structures), “β” family (containing β-sheet structures stabilized by disulfide bonds), α-hairpinin (with a motif formed by antiparallel α-helices that are stabilized by two disulfide bridges), and “αβ” family (having both “α” and “β” structures) [201]. On the other hand, peptides with extended/combined conformation [202].

AMPs are classified considering their similarity to protein sequence, cysteine motifs, and distinctive patterns of disulfide bonds, determining the peptide folding. Therefore, they are commonly grouped as thionins, defensins, heveins, knottins, lipid transfer proteins, and cyclotides. Thionins are a family of antimicrobial peptides with low molecular weight (about 5 kDa), rich in arginine, lysine, and cysteine residues. Their structure includes two antiparallel α-helices and an antiparallel double-stranded β-sheet with three or four conserved disulfide linkages. They are positively charged with a neutral pH. Their toxic effect was postulated to arise from the lysis of the membranes of attaching cells. However, the precise mechanism underlying toxicity remains unknown [203]. Plant defensins consist of three antiparallel β-sheets and an α-helix parallel to them. They possess a variety of biological functions, such as inhibiting microbial growth and inhibiting enzyme activity [204]. The hevein family consists of positively charged peptides of 29 to 45 amino acids, with abundant glycine (6) and cysteine (8–10) and aromatic residues. They have a coil-β1-β2-coil-β3 structure that occurs by variations with the secondary structural motif in the presence of turns in two long coils in the β3 chain. Hevein domains bind to chitin, which is their primary target; usually, their action modes include degradation and disruption of the cell wall and plasma membrane due to their hydrolytic action, causing extravasation of plasma particles, so heveins have good antifungal activity [201]. Plant knottins contain approximately 30 amino acids. Their antimicrobial activity has been attributed to alterations in functional components of the plasma membrane. The typical structure of knottins involves conserved disulfide bonds between multiple cysteine pairs, forming a cystine knot [204]. The lipid transfer proteins (LTPs) family comprises cationic proteins of approximately 70 and 90 amino acids with eight cysteine residues. They share a defining structural feature, a conserved inner hydrophobic cavity surrounded by α-helices. They bind to a wide range of lipids, including fatty acids, phospholipids, prostaglandin B2, lyso-derivatives, and acyl-coenzyme A. Plant LTPs inhibit bacterial and fungal pathogens’ growth by promoting pathogen membrane permeabilization [205]. Finally, cyclotides are ultra-stable peptides. They are around 30 amino acids and are disulfide-rich peptides from plants with a head-to-tail cyclic backbone and cystine knot arrangement of three conserved disulfide bonds [206].

Huan (2020) proposed that AMPs can also be classified based on amino acid-rich species, proline, tryptophan, arginine, histidine, and glycine-rich peptides [207,208]. Therefore, the data presented in Table 6 indicate that the most frequent residues were alanine, arginine, glycine, valine, and cysteine. Furthermore, an average isoelectric point of 8.5 and an average hydropathy of −0.15, showing the cationic and hydrophilic nature of most AMPs. Beyond these classical families of plant AMPs, recent studies have also pointed out that enzyme-derived cryptic peptides (cryptides) may represent an additional reservoir of antimicrobial molecules. For instance, ribosome-inactivating proteins (PD-L1/2) from *Phytolacca dioica* have been reported to release bioactive cryptides with antimicrobial and anti-biofilm activity [209]. Remarkably, these peptides exhibit structural plasticity, shifting between α-helix and β-sheet conformations depending on their environment (e.g., TFE, SDS, alginate, or LPS), directly influencing their bioactivity. More broadly, enzymes have been proposed as reservoirs of host defense peptides, underscoring the importance of considering cryptides as a relevant but underexplored source of plant-derived AMPs [210].

**Figure 2:**
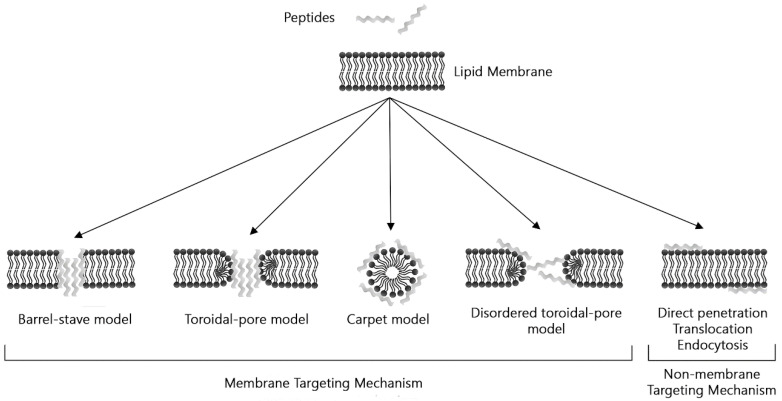
Schematic illustration of models of action by AMPs.

Antimicrobial mechanisms of these small amino acid fragments are as heterogeneous as their structure, and some are based on breaking the membrane to cause lysis of bacterial cells [226]. Cationic AMPs generally exhibit a balance between hydrophobic and positively charged amino acid residues, allowing them to adopt an amphipathic conformation, which allows greater interaction with negatively charged bacterial membranes, which helps promote their insertion [227]. There are four models of interaction between an AMPs and the cell membrane. In (I) Barrel-stave model, AMPs insert vertically into the plasma membrane to form transmembrane pores. Here, hydrophobic regions of AMPs align with lipid tails. In (II) Carpet model, peptides are adsorbed parallel to the lipid bilayer to cover the cell surface. Here, peptides disrupt the membrane in a detergent-like manner, breaking the lipid bilayer into separate micelles. (III) The toroidal pore model is an intermediate type between the carpet and the barrel. Finally, (IV) in the disordered toroidal pore model, the pore formation is more random and involves fewer peptides, but additional peptides must stabilize the opening (Figure 2) [203,204,207,228].

### 2.7. Relation Between Plant Peptides Structure and Its Antiviral Activity

Recent evidence highlights that some AMPs also present activity against various viruses, thus being called antiviral peptides (AVPs). Several properties may influence the antiviral activity of peptides, such as the topology, amino acid composition, charge, and many other chemical and structural characteristics. The overall biochemical features of AVPs are cationic and amphipathic characteristics and positive net charges. It has been observed that AVPs act primarily by directly inhibiting the viral particle, competing for the receptor on the target cell, and blocking its interaction/adsorption. However, they may also act at other levels of the viral cycle [229]. Miscellaneous AVPs target various steps in the viral cycle from receptor binding to replication and may be virucidal. In addition, some peptides can also be translocated into the cell cytoplasm and interact with intracellular targets, interfering with physiological and chemical functions, such as nucleic acid or protein synthesis [199]. We performed a bioinformatic analysis with the AVPs, as shown in Table 7. We observe that the most frequent residues in AVPs were cysteine, proline, glycine, isoleucine, and aspartic acid. In contrast, asparagine, proline, aspartic acid, isoleucine, and valine are found in more peptides. Likewise, we observed an average isoelectric point of 5.18 and an average hydropathy of −0.23.

To synthesize the information discussed in Section 2.1, Section 2.2, Section 2.3, Section 2.4, Section 2.5, Section 2.6 and Section 2.7, Table 8 summarizes the main structural and physicochemical features of plant-derived peptides associated with seven major biological activities. Table 8 integrates evidence on peptide length, net charge, secondary-structure tendencies, hydrophobicity patterns, and recurrent amino acids, highlighting general trends and mechanisms reported in the literature. This integrative overview provides a practical framework to connect peptide structural features with their observed bioactivities.

## 3. New Plant Sources for Bioactive Peptides

For a long time, legumes and cereals were the primary sources of bioactive peptides, particularly from legumes. They represent 27% of the world’s primary agricultural production, supplying around 15% of the protein worldwide [236]. However, obtaining peptides from legumes has become a concern, as it has been observed that these peptides may retain the allergenic sequence of various legume proteins [237]. As for cereal proteins, the trend of consuming gluten-free products has diminished their demand, even for gluten-free cereals such as maize, buckwheat, rice, millet, quinoa, etc., since it is known that they can be contaminated with gluten during processing, transportation, and handling [238]. For this reason, in recent years, peptides have been obtained from plant-derived sources such as leaves (e.g., *Moringa oleifera*, *Camelia sinensis*, *Spinaca oleracea*) or fruits (e.g., *Cucumis melo*, *Citrus* species, *Stenocereus pruinosus*), which have demonstrated bioactivities including antioxidant, antihypertensive, and anticancer effects, thus representing a safer alternative for consumers [239,240,241].

## 4. Current Scenario and Future Perspectives of Peptide-Based Drugs

Currently, the bioactive properties of peptides are often evaluated at non-physiological concentrations, which can lead to overestimating their antioxidant, anti-inflammatory, hypotensive, hypolipidemic, antimicrobial, and antiviral activities. For example, many *in vitro* studies report antioxidant activity at concentrations ranging from 0.1 to 1 mM. In contrast, physiological plasma concentrations after oral intake are typically in the low micromolar or even nanomolar range (≤10 µM). Similarly, antihypertensive peptides derived from food proteins are commonly tested at doses of 0.5–5 mg/mL, which far exceed the levels likely to be achieved systemically in vivo [242]. This discrepancy underscores the importance of validating bioactivities under conditions that mimic physiological environments. In this sense, future research should prioritize the use of physiologically relevant concentrations and incorporate pharmacokinetic studies addressing absorption, distribution, metabolism, and excretion (ADME) to assess these molecules’ bioavailability accurately. The application of in vivo models is also essential to determine therapeutic doses, safe administration periods, and potential toxicity profiles. Additionally, computational tools such as molecular docking and Density Functional Theory (DFT) simulations have become invaluable for screening thousands of peptide sequences, allowing researchers to identify candidates with higher chances of in vivo efficacy and pharmacological stability [242,243].

Beyond these aspects, other translational challenges also require attention. Purification remains a critical step, as many studies rely on different isolation and fractionation strategies—such as ultrafiltration and various chromatographic techniques—which makes results difficult to compare. The lack of standardized protocols for peptide production, purification, and characterization further complicates reproducibility across laboratories [244]. Moreover, peptide stability under physiological conditions is often compromised due to susceptibility to proteolytic degradation [245]. This highlights the need for innovative formulation strategies, such as microencapsulation, liposomes, or nanocarriers, which can improve stability, protect peptides from degradation, and enhance controlled release [246]. At the same time, the ability to target relevant tissues remains limited. Most bioactive peptides are studied in general systemic contexts, but specific delivery to organs such as the heart, liver, or brain will be essential for their therapeutic translation [247]. Recent work on peptide-based nanoparticles also emphasizes challenges related to stability, scalability, and tissue selectivity [248].

While bioactive peptides are generally considered safe, more systematic assessments of their safety profiles are required to confirm the absence of long-term or context-dependent adverse effects. Notably, evidence suggests that peptide activity can vary with factors such as dose, cellular context, or extracellular conditions, highlighting the need for deeper mechanistic studies under physiologically relevant scenarios [249].

These considerations highlight that while plant-derived peptides hold substantial promise as future therapeutic agents, further efforts in standardization, formulation, mechanistic elucidation, and safety assessment are necessary to bridge the gap between laboratory findings and clinical application. Together with the increasing consumer demand for plant-derived biopeptides, these strategies point toward a promising future for peptide-based drugs, provided that translational challenges are addressed through realistic experimental approaches [233,234].

## 5. Conclusions

Numerous diseases worldwide significantly impact the quality of life of large population groups. Consequently, searching for natural-origin molecules with therapeutic and preventive potential has gained considerable attention. In this context, peptides have emerged as promising agents against communicable and non-communicable diseases. Among these, plant-derived peptides stand out as an attractive option due to their vast botanical diversity, sustainability, and alignment with circular economy strategies. Compared to animal-derived peptides, they often present distinct structural and functional characteristics, such as lower allergenic potential and specific sequence–activity relationships. Recent scientific advances have established a clear relationship between the bioactivity of these molecules and their structural characteristics. This has underscored the importance of peptide purification and structural identification, which are now essential in studies evaluating their biological potential. These developments represent a significant step forward in peptide-based therapeutics. Nevertheless, critical challenges remain: the stability of peptides under physiological conditions, their bioavailability and tissue targeting, and the lack of standardized protocols for production and characterization continue to limit translation into real-world applications. Furthermore, depending on concentration, cell type, or extracellular environment, peptides may exert different or opposite effects, highlighting the need for deeper mechanistic studies under physiologically relevant conditions.

Integrating advanced purification methods, computational screening, and in vivo validation will be crucial to fully exploit the therapeutic promise of plant peptides. If these barriers are addressed, plant-derived bioactive peptides could evolve from experimental findings into reliable components of functional foods and peptide-based drugs, ultimately contributing to both human health and sustainable innovation.

## Figures and Tables

**Table 1:** Summarization of plant peptides with antioxidant activity.

Plant Source (Common Name)	Obtaining Method	Peptides Sequences	Activity	Reference
Apricot	Alcalase	SHNLPILR and SEAGVTE	Hydroxyl radical and superoxide anion radical scavenging capacities of 60 and 50% (1 mg/mL) and DPPH of 25% (1 mg/mL).	[28]
Asparagus	Alcalase	FAPVPFDF, MLLFPM * and FIARNFLLGW	DPPH scavenging Activity (EC_50_ of 4.14 μmol/L).	[29]
Basul	Alcalase	10 peptides (CCGDYY * among them)	ABTS and ORAC scavenging Activity (IC_50_ of 1.18 and 3.61 μmol TE/μmol).	[30]
Bitter bean	Alcalase	29 peptides	DPPH and reducing power of 2.9 mg GAE/g and 11.7 mM, respectively.	[31]
Cress	Alkaline protease and neutral protease	GRVGSSSC, GRAGGSYM, GHPNFKLNCSGG, GTKSCKA, ASSNARDMI, TAGGCYIPI, and KNCALQ	DPPH, OH^−^, O_2_^−^, and ABTS scavenging activities of 89.2, 26.3, 40.6, and 42.9%, respectively (0.1 mg/mL).	[32]
Cherrie	Alcalase	14 peptides	ABTS, FRAP, and Hydroxyl scavenging activity of 40, 35, and 20%, respectively.	[33]
Cherries	Thermolysin	22 peptides	ABTS, FRAP, and Hydroxyl scavenging activity of 55, 45, and 30%, respectively.	[33]
Chickpea	Neutrase	LTEIIP	DPPH and hydroxyl radicals scavenging activity (IC_50_ of 0.24 and 0.57 mg/mL, respectively).	[34]
Chickpea	Alcalase	NRYHE	DPPH, Hydroxyl, and superoxide free radicals scavenging activity of 50, 80, and 60%, respectively.	[35]
Chickpea	Pepsin and pancreatin	ALEPDHR *, TETWNPNHPEL, FVPH, and SAEHGSLH	50 CAA units.	[26]
Chickpea	Alcalase	DHG and VGDI *	DPPH scavenging activity of 67% at 200 μg/mL	[36]
Chickpea	Alcalase and flavorzyme	RQSHFANAQP	DPPH and Hydroxyl radical scavenging activity of 64.9 and 95.8%, respectively (5 mg/mL)	[37]
Corn	Alcalase and flavouryzme	CSQAPLA *, YPKLAPNE, and YPQLLPNE	DPPH radical and superoxide anion radical (IC_50_ of 0.116 and 0.39 mg/mL, respectively).	[38]
Corn	Alcalase	MGGN, MNN * and MEN	CAA of 1213.79 μmol of QE/100 mmol	[39]
Corn	Alcalase and trypsin	MI/LPP	DPPH scavenging activity (IC_50_ of 220 μg/mL).	[40]
Corn	Alcalase	AGI/LPM * and HAI/LGA	Scavenges hydroxyl radicals by 79.41% (10 mg/mL)	[41]
Corn	Alkaline protease and flavourzyme	LPF, LLPF, and FLPF *	DPPH and ABTS radical scavenging activity (IC_50_ of 1.51 and 2.83 mM, respectively).	[42]
Corn	Alcalase	YA and LMCH *	ABTS, DPPH, and O_2_ of 25, 15, and 30%, respectively (1, 10, and 10 mg/mL, respectively)	[43]
Cottonseed	Microbial fermentation	YSNQNGRF	DPPH, ABTS, and hydroxy radical scavenging activity (EC_50_ of 0.49, 2.05, and 2.21 mg/mL).	[44]
Hemp	Pepsin and pancreatin	WVYY * and PSLPA	DPPH scavenging activity of 67% (0.5 mg/mL).	[45]
Hemp	Alcalase	NHAV and HVRETALV	Protective effects against cell death and oxidative apoptosis.	[46]
Hemp	Protamex	YGRDEISV and LDLVKPQ	ABTS, FE^2+^ chelating activity, and hydroxy radical scavenging activity of 52.3, 52.9, and 50.9%, respectively (0.4 mg/mL).	[47]
Hemp	Alcalase	LLY, LLR, IR, TY, VY, LH, EL, LK, AY, H, R and F.	ABTS, DPPH, FCA, and FRAP (IC_50_ of 0.5, 1, 1.2, and 1 mg/mL, respectively).	[48]
Jackfruit	Trypsin	VGPWQK	ABTS EC_50_ of 1 mg/mL	[49]
Jujube	Trypsin	VGQHTR * and GWLK	ABTS, DPPH, and chelating activity of 30, 20, and 15%, respectively (0.3 mg/mL).	[50]
Korean Pine	Alcalase	KWFCT and QWFCT *	ABTS scavenging activity of 74.9% (2 mg/mL), CAA of 916.32 µmol of QE/100 g.	[51]
Lentil	Savinase	LLSGTQNQPSFLSGF, NSLTLPILRYL * and TLEPNSVFLPVLLH	ORAC 1.432 μmol TE/μmol.	[52]
Lotus	Flavourzyme	16 peptides	DPPH and H_2_O_2_ scavenging (EC_50_ of 2.9 and 16.1 mg/mL, and reducing power activity of 8.0 mg/mL, respectively)-	[27]
Moringa	Pepsin and pancreatin	20 peptides	DPPH and ABTS of 45.70 and 93.09%, respectively (1.33 mg/mL), ORAC 1.27 mM TE/g.	[53]
Mulberry	Neutrase	SVL, EAVQ, and RDY *	DPPH and ABTS scavenging activity of 45 and 100%, respectively (0.4 mg/mL), CAA of 2204 μM QE/100 g.	[54]
Mung bean	Pepsin and pancreatin	MD, QSA, EW, LGW, KK, SVP, and DVAF	ABTS, ORAC, and iron chelating activity of 581.3 ascorbic acid, 127 TE, and 4843.5 EDTA equivalent, respectively (1 µM).	[55]
Mung Bean	Thermolysin	KK, DM, S, Y and W	ABTS, ORAC, and iron chelating activity of 646.2 ascorbic acid, 127 TE, and 5161.7 EDTA equivalent, respectively (1 µM).	[55]
Mung bean	Pancreatin	10 peptides (LLGIL * among them)	DPPH and hydroxyl radical neutralizers of 81.27% (4 mg/mL) and EC_50_ of 0.37 mM, respectively.	[56]
Mung Bean	Bromelain	CTN, HC, CGN, and CSGD	DPPH radical scavengers (EC_50_ values of 0.30, 0.29, 0.28, and 0.30 mg/mL, respectively).	[57]
Mungbean	Neutral protease	WN, WGN, LY, AW, RGWYE *, FW, GVPFW, and WLF	DPPH, ABTS, and OH^−^ radical scavenging activities of 60%, 35%, and 30%, respectively.	[58]
Palm	Papain	YLLLK, YGIKVGYAIP, GGIF *, GIFE, WAFS, GVQEGAGHYALL, WAF, AWFS, and LPWRPATNVF	DPPH scavenging Activity (IC_50_ of 0.02 μM).	[59]
Pearl millet	Trypsin	SDRDLLGPNNQYLPK	DPPH, ABTS, Fe^2+^ chelating ability, and hydroxyl assay of 67.66, 78.81, 51.20, and 60.95%, respectively.	[60]
Peony	Alcalase	FSAP, PVETVR, QEPLLR, EAAY * and VLRPPLS	ABTS and hydroxyl radical-scavenging activity of 98.5 and 61.9%, respectively (0.5 mg/mL).	[61]
Perilla	Alkaline protease	YL and FY *	Inhibition of lipid peroxidation in rat liver (IC_50_ of 6.81 mg/mL), 0.05 mg/mL elevated H_2_O_2_ exposed cell ratios from 44.3 to 57.8%.	[62]
Perilla	Trypsin	ISPRILSYNLR	DPPH and ABTS scavenging activity of 48.13 and 78.14%, respectively (0.1 mg/mL).	[63]
Rice	Papain, flavourzyme, and protamex	RPNYTDA, TSQLLSDQ, TRTGDPFF * and NFHPQ	DPPH, ABTS, and FRAP scavenging activity (IC_50_ of 0.446, 0.658, and 0.235 mg/mL, respectively).	[64]
Rice	Trypsin	YSK	DPPH and reducing power (IC_50_ of 0.15 and 0.125 mg/mL, respectively).	[65]
Rice	Trypsin	91 peptides	ORAC 4.07 μmol TE/mg.	[66]
Rice	Alcalase	SGDWSDIGGR *, DFGSEILPR, GEPFPSDPKKQLQ, and GEKGGIPIGIGK	ORAC and DPPH of 41.57 μmol TE/g and 29.41%.	[67]
Sorghum	Protease-Purazyme	11 peptides	ABTS of 6.10%	[68]
Soybean	Alcalase	FDPAL	The scavenging rate on superoxide anion and hydroxyl radical was 75% and 80%, respectively. (2 mM)·500 µM elevated H_2_O_2_ exposed HeLa cells’ survival rate.	[69]
Soybean	Pepsin and pancreatin	28 peptides (FSREEGQQQGEQ * among them)	ROS production by Caco-2 was lowered by half.	[70]
Sweet potato	Alcalase	TYQTF, SGQYFL, YMVSAIWG, YYIVS * and YYDPL	OH^−^ scavenging activity of 74.52% (1.0 mg/mL).	[71]
Tomato	Alcalase	29 peptides	DPPH scavenging activity of 60.36% (1 mg/mL).	[72]
Walnut	Alcalase	ALWPF and PLRWPF	Zebrafish embryo oxidative damage model values of 50 μg/mL and 30 μg/mL, respectively.	[73]
Wheat	Alcalase	CGFPGHC, QAC, RNF, SSC, and WF	TEAC and superoxide anion radical scavenging activity of 4760 μmol TE/g and 43.72%, respectively (5 mg/mL).	[74]

* This symbol was placed when the reported activity is associated with a particular peptide sequence.

**Table 2:** Summarization of plant peptides with antiproliferative activity.

Plant Source (Common Name)	Obtaining Method	Peptides Sequences	Activity	Reference
Bean	Pepsin and pancreatin hydrolysis	GLTSK, LSGNK, GEGSGA *, MPACGSS and MTEEY	Cell cycle arrest (G1) and Apoptosis induction (loss of mitochondrial membrane potential) in HCT116 cells (IC_50_ of 134.6 μM).	[88]
Chickpea	Alcalase hydrolysis	ADLPGLK	Cytotoxicity to Ishikawa cells (IC_50_ of 101.5 μg/mL), inhibition of Ishikawa cells proliferation by 68.9% (500 μg/mL), and activation of caspase-3 and cell cycle arrest at S and G2.	[87]
Common bean	Naturally occurring	KTYENLADTY KGPYFTTGSH DHYKNKEHLRSGRMRDDFF	Cytotoxicity to L1210 and MBL2 cells (IC_50_ of 4.0 and 9.0 μM, respectively).	[89]
Ginger	Pepsin hydrolysis	RALGWSCL	Cytotoxicity to NB4 cells (IC_50_ of 600 μg/mL) and upregulation of caspase 3, 8, and 9.	[79]
Lentil	Trypsin hydrolysis	27 peptides	Cytotoxicity to PC3 cells (IC_50_ of 0.96 mg/mL).	[90]
Lentil	Naturally occurring	Bowman-Birk trypsin inhibitor	Cytotoxicity to HT29 cells (IC_50_ of 32 μM).	[91]
Lima Bean	Naturally occurring	KTCENLATY and RGPCF	Antiproliferative activity BEL-7402 and SHSY5Ycells	[92]
Maize	Alcalase hydrolysis	EDLPGCPRE, LTKYNIT, DFGGGHPDPN, EDIIPF, MMAAAAGAG, DAVLQTLA, AAFVVAGT and MEVAMAM	Cytotoxicity to HepG2 cells (IC_50_ of 8.9 μg/mL).	[93]
Narcissus	Naturally occurring	GNNNLTSSQQQQIQFILQQI	Inhibited leukemia cells’ proliferation by 100% (80 μM)	[94]
Nut	Trypsin hydrolysis	AYRNRYRRQY, RYEQRPRT, and LPTSEAAKY	Inhibited HeLa cell proliferation by 19% (50 μg/L).	[86]
Pea	Naturally occurring	Bowman-Birk trypsin inhibitor	Cytotoxicity to HT29 cells with an IC_50_ of 31 μM.	[82]
*Pombalia calceolaria*	Naturally occurring	GLPCAESCVFIPCTITAILGCSCRDRVCYD *, GIPCAESCVFIPCVTAILGCSCKDRVCYN and GIPCGECVWIPCISSAIGCSCKNKVCYRN	Cytotoxicity to MDA-MB-231 cells (IC_50_ of 1.8 μM).	[95]
Quinoa	Pepsin and pancreatin hydrolysis	SENIDDPS, DVYSPEAG, EAGRLTTL, IRAMPV, IFQEYI, SFFVFL, RELGEWGI GGLGDVLGGLP	Cytotoxicity to Caco-2, HT-29, and HCT-116 cells (IC_50_ of 0.256, 0.195, and 0.193 mg/mL, respectively).	[96]
Quinoa	Trypsin hydrolysis	FHPFPR, NWFPLPR, and HYNPFPG	Inhibited Ishikawa cells proliferation by 53.93% (8 g/L) and upregulation of caspase-3.	[96]
Rapeseed	Fermentation with *Bacillus subtilis* and *A. elegans* Plus, neutral protease hydrolysis	WTP	Inhibited HepG2 cells proliferation by 58.86% by increasing p53 and BAX expression and decreasing BCL-2 expression (800 μg/mL)	[97]
Rapeseed	Naturally occurring	NDGNQPL	Cytotoxicity to HepG2 cells (IC_50_ of 1.56 mmol/L) and cell cycle arrest at G0/G1.	[98]
Rice	Alcalase and Gastrointestinal juices hydrolysis	EQRPR	Inhibited Caco-2, MCF-7, and HepG-2 cells growth by 84%, 80%, and 84%, respectively.	[99]
Sacha inchi	Alkaline and neutral proteases	LLEPDVR, ALVEKAKAS and TGDGSLRPY	Cytotoxicity to HepG2 cells (IC_50_ of 109 μg/m).	[84]
Soybean	Alcalase hydrolysis	L/I-VPK	Cytotoxicity to HepG2, MCF-7 and HeLa cells (IC_50_ of 0.22, 0.15, and 0.32 μM, respectively).	[100]
Soybean	Naturally occurring	Vglycin	Promoted apoptosis and cell cycle arrest at G1/S in CT-26, SW480, and NCL-H716 (IC_50_ values of 4.21, 3.68, and 3.62 μmol/L, respectively). 38% inhibition of colon cancer in mice at 20 mg/kg/Day/21 days.	[101]
Soybean	Pepsin and pancreatin hydrolysis	15 peptides	Cytotoxicity to Caco-2, HT-29, and HCT-116 cells (IC_50_ of 10.3, 14.9, and 15.2 mg/mL, respectively).	[102]
Soybean	Alcalase, pepsin, and pancreatin hydrolysis	158 amino acid residues peptide	Inhibited Caco-2 and HCT-116 cells proliferation by 80% (700 μg/mL).	[103]
Soybean	Pepsin, trypsin, and chymotrypsin hydrolysis	CSDMRLNSCHSA, LSYPAQC, CYEPCKPSEDDKEN and PCKPSEDDKEN	Cytotoxicity to HT-29 cells (IC_50_ of 0.29 mg/mL).	[104]
Sweet potato	Naturally occurring	AASTPVGGGR and RLDRGQ	Inhibited Panc-1 cells’ proliferation by 55% by promoting apoptosis through the mitochondrial apoptotic pathway (100 μM)	[105]
Walnut	Papain hydrolysis	CTLEW	Cytotoxicity to Caco-2 and HeLa cells (IC_50_ of 0.65 and 0.60 mg/mL, respectively).	[86]
Walnut	Papain hydrolysis	CTLEW	Cytotoxicity to Caco-2, HeLa, and MCF-7 cells (IC_50_ of 0.65, 0.60, and 0.449 mg/mL, respectively).	[86]

* This symbol was placed when the reported activity is associated with a particular peptide sequence.

**Table 3:** Summarization of plant peptides with ACE-inhibitory activity.

Plant Source (Common Name)	Obtaining Method	Peptides	ACE Inhibitor Activity	Reference
Hempseed	Pepsin, trypsin, and pancreatin	SHLNWVCIFLGFHSFGLYI, QIQFEGFCRF, and IPDKANLGFRFP	Determined as an ACE-inhibitor using the PeptideRanker and BIOPEP	[119]
Huangqi root	Aqueous extraction	LVPPHA	IC_50_ of 414.88 μM	[116]
Longan seeds	Pepsin and pancreatin	ETSGMKPTEL and ISSMGILVCL	IC_50_ of 0.003 mg/mL	[120]
Moringa	Flavourzyme,	IPPAYSK, ILVDR *, FFFPK and LLDPR	IC_50_ of 81.25 μM	[111]
Moringa	Alcalase	LGFF and GLFF	IC_50_ of 0.29 and 1.88 mM, respectively	[121]
Olive	Alcalase, thermolysin, neutrase, PTN, and flavourzyme	LTPTSN, LVVDGEGY, and FDAVGVK	IC_50_ of 0.029 mg/mL	[122]
Stink bean seeds	Alcalase	ASPAPAGLSYCVPEVDPLLLRAEDKLDLSDL	51.7–80.2% inhibition	[123]
Plum	Alcalase, thermolysin, flavourzyme, and protease P.	MLPSLPK, HLPLL, NLPLL, HNLPLL, KGVL, and HLPLLR	IC_50_ of 0.5 mg/mL	[124]
Rice	Neutrase	FDGSPVGY * and VFDGVLRPGQ	IC_50_ of 0.079 mg/mL	[125]
Soybean	Alkaline proteinase, papain, trypsin, pepsin, and pancreatin	LLPLPVLK, WLRL, SWLRL and MLPVMR	51.43% inhibition	[126]
Soybean	Alkaline protease, flavored protease, papain, trypsin, and chymotrypsin	GKGLW and GDGLKW *	IC_50_ of 33.98 μM	[115]
Soybean	Alcalase	LY *, YVVF, LVF, WMY, LVLL, and FF	IC_50_ of 0.53 μM	[127]
Soybean	Alcalase and Flavourzyme	ALKPDNR, VVPD, NDRP, and NDTP	IC_50_ of 148.28 μg/ml	[118]

* This symbol was placed when the reported activity is associated with a particular peptide sequence.

**Table 4:** Summarization of plant peptides with hypolipidemic activity.

Plant Source (Common Name)	Obtaining	Peptides	Activity	Reference
Amaranth	Pepsin, trypsin, and pancreatin hydrolysis	GGV, IVG/LVG, VGVI/VGVL	4 μg/mL inhibited HMG-CoA reductase by 45%.	[132]
Bean	Pepsin and pancreatin hydrolysis	LVTTTVDL, QTSTPLFS, VELVGPK, TRGVLV	700 mg/kg/day for nine weeks in BALB/c mice decreased lipid profile, total cholesterol, triglycerides, and HDL-c levels.	[133]
Brewer’s spent grain	Purazyme and flavourzyme hydrolysis	WNIHMEHQDLTTME, DFGIASF, LAAVEALSTNG	0.4 mg/mL inhibited cholesterol esterase by 35% and 1.4 mg /mL inhibited pancreatic lipase by 80%.	[134]
Coffee	Alcalase, pepsin, pancreatin, and thermolysin hydrolysis	79 peptides	Reduced *in vitro* micellar cholesterol solubility by 32%.	[135]
Cowpea	Pepsin and pancreatin hydrolysis	11 peptides	35 μg/mL reduced cholesterol micellar solubilization by 71.7% and 50 μg/mL inhibited HMGCoAR by 57.1%.	[136]
Hemp seed	Pepsin hydrolysis	90 peptides	1.0 mg/mL inhibited HMGCoAR by 80%, up-regulated the LDLR protein levels by 63% and increased the LDL-uptake in HepG2 cells.	[137]
Hemp seed	Trypsin	20 peptides	Reduced serum total cholesterol, triglycerides, and low-density lipoprotein cholesterol by 28, 34, and 40% in high-fat-induced mouse.	[138]
Lupin	Pepsin hydrolysis	YDFYPSSTKDQQS	100 μM inhibited HMGCoAR by 87.4%, modulated the cholesterol metabolism in HepG2 cells, improved the low-density lipoprotein levels by 50.5% and increased the receptor protein levels by 37.8% via SREBP-1 activation.	[139]
Lupin	Trypsin hydrolysis	GQEQSHQDEGVIVR	Inhibited HMGCoAR, with an IC_50_ of 99.5 μM, 00 µM, enhanced the LDLR protein levels by 43.4%, reduced the PCSK9^D374Y^ protein level by 41.2% and decreased the HNF-1α level by 19.8% in HepG2 cells.	[140]
Lupin	Trypsin and pepsin hydrolysis Synthesized	YDFYPSSTKDQQS, LILPKHSDAD, LTFPGSAED, LTFPG	Produced a fast and efficient decrease in systolic blood pressure (−37 mmHg after 2 h).	[141]
Lupin	Trypsin and pepsin hydrolysis	LILPKHSDAD, LPKHSDAD, YDFYPSSTKDQQS, LTFPGSAED	Inhibited HMGCoAR (IC_50_ of 175.3 µM), increased LDLR expression, and decreased the PCSK9 production of HepG2 cells.	[142]
Olive seed	Alcalase hydrolysis	130 peptides	Reduced cholesterol micellar solubility by 49%, bound to 10% of total bile acid, and inhibited cholesterol esterase and HMGCoA enzymes by 30% and 15%, respectively.	[143]
Pinto bean	Protamex^®^	PPHMLP, PLPTGAGP, PPHMGGP, PLPLHMLP, LSSLGMGSLGALPVCM	4.59 μM inhibited pancreatic lipase by 87%.	[144]
Rapeseed	Alcalase	EFLELL	Decreased total cholesterol and triglyceride by 46% and 32%, respectively, at a concentration of 2 mM	[145]
Rice	Pepsin and trypsin hydrolysis	GEQQQQPGM	100 mg/body weight for 30 days reduced the atherogenic index.	[146]
Rice	N/A	IIAEK	300 mg/kg body weight/day reduced total serum cholesterol, LDL-cholesterol, and the atherogenic index.	[147]
Soybean	Trypsin and pepsin hydrolysis	IAVPGEVA, IAVPTGVA, LPYP	10 μM inhibited the activity of HMGCoAR and modulated cholesterol metabolism in HepG2 cells.	[148]
Soybean	Pepsin and trypsin hydrolysis	140 peptides	0.5 mg/mL inhibited HMGCoAR by 76.9% and Increased LDLR protein levels by 63.0% in HepG2 cells.	[149]
Soybean	Trypsin and pepsin hydrolysis	YVVNPDNDEN, YVVNPDNNEN	250 μM inhibited HMGCoAR by 80% and increased the LDL uptake by 2-fold in HepG2 cells.	[150]
Tea	Pepsin	FLF, IYF, and QIF	Inhibited pancreatic lipase and cholesterol esterase (IC_50_ values of 0.153 and 0.549 mg/mL, respectively).	[131]

N/A = Not available.

**Table 5:** Summarization of plant peptides with hypoglycemic activity.

Plant Source	Obtaining	Peptides	Activity	Reference
Bean	α-amylase, pepsin, and pancreatin hydrolysis	INEGSLLLPH, FVVAEQAGNEEGFE, INEGSLLLPH, SGGGGGGVAGAATASR, GSGGGGGGGFGGPRR, GGYQGGGYGGNSGGGYGNRG, GGSGGGGGSSSGRRP, GDTVTVEFDTFLSR,	α-amylase inhibitory activity (IC_50_ of 1.94 mg/mL)	[164]
Bean	Pepsin and pancreatin hydrolysis	22 peptides	Inhibited DPP-IV (IC_50_ 0.03 mg/mL)	[165]
Bean	Alcalase and bromelain hydrolysis	40 peptides	Inhibited α-amylase (50%), α-glucosidase (76.4%), and DPP-IV (55.3%)	[166]
Bittermelon	N/A	RVRVWVTERGIVARPPTIG.	600 mg/day/ 3 Regulated the blood glucose of diabetic patients and reduced the glycated hemoglobin by 7.4%.	[167]
Bittermelon	Naturally occurring	GHPYYSIKKS	1 mg/kg significantly reduced blood glucose levels (7.68 mmol/L) in an in vivo model	[168]
Bittermelon	Naturally occurring	H-GHPYYSIKKS-OH	In a murine model, 1 mg/kg showed anti-hyperglycemic effects and lowered blood glucose levels.	[169]
Bittermelon	Pepsin and pancreatin hydrolysis	SRCQGKSSWPQLVGSTGAAAKAWIERENPRVRAVIIKV, GSGATKDFRCDRVRVWVTERGIVARPPTIG, SRCQGKSSWPQLVGSTGA, GAAAKAWIERENPRVRAVI, VIIKVGSGATKDFRCDRVR, RVRVWVTERGIVARPPTIG, DFRCDRVRVWVTERGIVARPPTIG, VTERGIVARPPTIG	5 nmol/kg displayed a hypoglycemic activity *in vitro* and in vivo	[170]
Black bean	Alcalase, Trypsin, proteinase K, Flavourzyme, Thermolysin, Pepsin, Papain, and Chymotrypsin hydrolysis	34 peptides	1 mg/mL inhibited DPP-IV, α-amylase, and α-glucosidase by 96.7, 53.4, and 66.1%, respectively	[171]
Brewer’s spent grain	Alcalase, ultraFlo, shearzyme, and flavourzyme hydrolysis	IPY, LPY, IPLQP, LPLQP, APLP, VPIP, IPVP *, PLVP	DPP-IV inhibitory activity with an IC_50_ of 38.67 μM	[172]
Chickpea	Pepsin/pancreatin and bromelain hydrolysis	32 peptides	Had affinity for DPPV-IV, α-amylase, and α-glucosidase active sites.	[173]
Chickpea	Enzymatic hydrolysis (not specified)	LLR, FH, RQLPR, KGF, and NFQ	Inhibited α-glucosidase increased the viability of insulin-resistant cells and the glucose consumption rate.	[174]
Hemp	Alkaline protease	TGLGR, SPVI, FY, and FR	Inhibited α-glucosidase by 70.02% (20 µL of the hydrolysate).	[175]
Lupin	Trypsin and pepsin hydrolysis	LTFPGSAED	Inhibited DPP-IV (IC_50_ of 207.5 µM)	[176]
Mulberry	N/A	WGVENAATYFWQTV	α-amylase and α-glucosidase inhibitory activity (IC_50_ of 16.25 and 12.56 μg/mL, respectively)	[177]
Oat	Trypsin hydrolysis	LQAFEPLR, EFLLAGNNK, LQAFEPLR, EFLLAGNNK.	Inhibited DPP-IV (IC_50_ 141.7 μM) and modulated its expression in Caco-2 cells.	[178]
Oat, buckwheat, and highland Barley	Alcalase and trypsin hydrolysis,	35 peptides	Inhibited DPP-IV (IC_50_ from 0.13 to 8.15 mg/mL)	[179]
Orange seed	Porcine pepsin, trypsin, and chymotrypsin hydrolysis	63 peptides	20 µL Inhibited α-amylase and α-glucosidase by 41.7 and 57%, respectively	[180]
Orange seed	Pepsin, trypsin, and chymotrypsin hydrolysis	978 peptides	0.1 and 1 mg/mL inhibited α-amylase and α-glucosidase by 42.35 and 45.39%, respectively	[181]
Pinto bean	Protamex^®^	PPHMLP, PLPTGGAGP, PPHMGGP, PLPLHMLP, LDDLGMGSLGALPVCM	100 μg/mL Inhibited α-amylase (IC_50_ of 0.31 mM)	[182]
Potato	Alcalase hydrolysis	DIKTNKPVIF	50 mg/kg/day lowered glucose and HbA1c levels in diabetic and non-diabetic mice.	[183]
Quinoa	α-chymotrypsin, Pronase E, and Bromelain hydrolysis	136 peptides	Inhibition of DPP-IV and α-glucosidase (IC_50_ of 1.12 and 1.86 mg/mL, respectively)	[184]
Quinoa	Pepsin and pancreatin hydrolysis	20 peptides	250 μM inhibited DPP-IV, α-amylase, and α-glucosidase by 17, 6.86, and 55.85%, respectively	[185]
Soy, lupin, and quinoa	Subtilisin, trypsin, and flavourzyme hydrolysis	20 peptides	Inhibited DPP-IV (IC_50_ 1.16 mg/mL)	[186]
Soybean	Pepsin and trypsin hydrolysis	140 peptides	2.5 mg/mL reduced the DPP-IV activity by 31.4%.	[149]
Soybean	Pepsin and pancreatin, hydrolysis	12 peptides	Inhibited DPP-IV (IC_50_ of 0.91 mg/mL), α-amylase (IC_50_ of 1.70 mg/mL), and α-glucosidase (IC_50_ of 3.73 mg/mL).	[187]
Soybean	Naturally occurring	VSCNGVCSPFEMPPCGSSACRCIPYGLVVGNCRHPSG	40 μg/[g·d] normalized fasting glucose levels in diabetic rats	[188]
Soybean	Trypsin hydrolysis	GSA, GAL	α-glucosidase inhibitory activity (IC_50_ 0.049 mg/mL) 3.52 mg/mL reduced fasting blood glucose in diabetic mouse by 42%	[189]
Soybean	Alkaline proteinase, papain, and trypsin hydrolysis	LLPLPVLK, SWLRL, WLRL *	α-glucosidase inhibitory effect (IC_50_ 162 μmol/L) 2.73 mg/mL inhibited the enzyme DPP-IV in 40.85%	[126]
Soybean	Trypsin and pepsin hydrolysis	IAVPTGVA	Inhibited DPP-IV (IC_50_ of 223.2 µM)	[176]
Tea	Fermentation	AADTDYRFS and AGDGTPYVR	Inhibited α-glucosidase with IC_50_ of 0.820 and 3.942, respectively. Increased expression levels of MDM2, IRS-1, Akt, PI3k and GLUT4.	[190]

N/A = Not available. * This symbol was placed when the reported activity is associated with a particular peptide sequence.

**Table 6:** Summarization of plant peptides with antimicrobial activity.

Plant Source (Common Name)	Obtaining	Peptides Sequences	Target Pathogen	Reference
Alfalfa	Pepsin	MDN, TMW, CVQ, AFR, ELAAAC, ILAAF, GNAPGAVA, LRDDF, ALRMSG, RDRFL, EYLIRKGWI, IEKDRSRGIF	*Escherichia coli*, *Micrococcus luteus*, *Bacillus subtilis*, and *Listeria innocua*.	[211]
Angel’s trumpets	Naturally occurring	RHCESQSQRFKGTCLSEKNCASVCETEGFSGGDCRGLRRRCFCTRPC	*Staphylococcus epidermidis*.	[212]
Barrel medic	Naturally occurring	RNGCIVDPRCPYQQCRRPLYCRRR	*Sinorhizobium meliloti*	[213]
Bitter tomato	Naturally occurring	MKTIQGQSATTALTMEVARVQA	*Rhizoctonia solani* and *Colletotrichum gloeosporioides*.	[214]
Chickweed	Naturally occurring	VDPDVRAYCKHQCMSTRGDQARKICESVCMRQD	*Alternaria alternata*, *Aspergillus niger*, *Bipolaris sorokiniana*, *Botrytis cinérea*, *Fusarium oxysporum*, *Fusarium solani*, *Phytophthora infestans* and *Pythium ultimum*.	[215]
Chili pepper	Naturally occurring	N-terminal: AVTXGQVDANLAPXV	*Colletotrichum lindemunthianum* and *Candida tropicalis*.	[216]
Cowpea	Naturally occurring	N-terminal sequence: KTCMT- Nested in Cp-thionin II	*Fusarium culmorum.*	[217]
Cowpea	Alcalase	CW, SC, WS, CR, WC, LA, GP, NV and RG	*Listeria monocytognes*, *Listeria innocua*, *Staphylococcus aureus*, *Sreptococcus pyogenes*, *Klebsiella pnemoniae*, *Pseudomonas aeruginosa*, *Escherichia coli* and *Salmonella typhimurium*.	[208]
Ground Bean	Naturally occurring	N-terminal sequence: KTCENLADTY	*Botrytis cinerea*, *Fusarium oxysporum*, *Mycosphaerella arachidicola*, *Escherichia coli*, *Proteus vulgaris*, *Mycobacterium phlei* and *Bacillus megaterium*.	[218]
Phytolocca dioica	Ribosome inactivating protein PD-L1/2 (precursor)	2 peptides	*Planktonic bacterial* cells	[209]
Potato	Acid digestion	AVCENDLNCC	*Candida albicans*.	[219]
Rice	Pepsin	LRRHASEGGHGPHW FSKGVQRAAF EKLLGKQDKGVIIRA SSFSKGVQRAAF SSFSKGVQRAAF	*Porphyromonas gingivalis* and *Candida albicans*,	[220]
Rice	Naturally occurring	RRLMAAKAESRK	*Escherichia coli* and *Aggregatibacter actinomycetemcomitans*.	[221]
Rice bran	Bromelain	KVDHFPL	*Listeria monocytogenes*.	[222]
Sea onion	Naturally occurring	QIPLTGAHSIIGRA IPLSGPNAVIGRA	*Staphylococcus aureus* and *Pseudomonas aeruginosa*.	[223]
Soy	Synthesized	PGTAVFK IKAFKEATKVDKVVVLWTA	*Listeria monocytogenes* and *Pseudomonas aeruginosa*.	[224]
Soybean	Naturally occurring	PRPIPFPRPQP	*Escherichia coli*, *Staphylococcus aureus*, *Staphylococcus saprophyticus*, *Aeromonas hydrophila*, *Vibrio parahaemolyticus*, and *Salmonella enterica*.	[225]

**Table 7:** Summarization of plant peptides with antiviral activity.

Plant Source (Common Name) *	Peptides Sequence	Target Pathogen	Reference
Catechu	DHVTPDIAYNPRTYM DHVTPDIAYNPWAYF	Dengue virus	[230]
Chili pepper	Cb-1: GFPFLLNGPDQDQGDFIMFG Cb-1′: GFKGEQGVPQEMQNEQATIP	Pepper yellow mosaic virus	[231]
Devil’s tree	CRPYGYRCDGVINQCCDPYHCTPPLIGICL CRPYGYRCDGVINQCCDPYRCTPPLIGICL CVPRFGRCDGIINQCCDPYLCTPPLVGICT CVPQYGVCDGIINQCCDPYYCSPPIYGHCI	Infectious bronchitis virus Dengue virus	[232]
Ground Bean	N-terminal sequence: KTCENLADTY	Human immunodeficiency virus	[218]
*Oldenlandia affinis*	CGETCVGGTCNTPGCTCSWPVCTRNGLPV	Human immunodeficiency virus	[233,234]
Sorghum	-	Herpes simplex virus type 1	[235]

* All peptides were naturally occurring.

**Table 8:** Summary of structural and physicochemical features of plant-derived bioactive peptides by biological activity.

Biological Activity	Peptide Length (Amino Acids)	Net Charge	Secondary Structure	Key Hydrophobicity Traits	Key Amino Acids	Mechanism of Action	Reference
Antioxidant	2–21 (66% are 2–10)	0	Low content of random-coil and α-helix	Hydrophobic residues enhance membrane interaction	E, D, G, A, L, F, Y, H, M, C	Radical scavenging (DPPH, ABTS, ORAC); metal chelation; lipid peroxidation inhibition; activation of endogenous antioxidant enzymes	[41,51,62,78]
Antiproliferative	3–158 (63% are 3–10)	≥+1	β-pleated sheet peptides	>30% hydrophobic content enhances membrane interactions	E, L, S, F, A, K, R, P, W, G	Membrane pore formation; apoptosis (mitochondrial); autophagy-like death; interaction with HDAC1, MDM2; NK cell activation	[79,87,98]
Ace-inhibitory	2–10	0	β-turn/random-coil common; small, compact motifs	Hydrophobic pockets	L, I, P, Y, F, W, D, E	Competitive, noncompetitive, and uncompetitive inhibition	[109,112,118]
Hypolipidemic	2–20		Flexible; no strict fold requirement	≥4 hydrophobic residues promote lipid interactions	K, T, V, E, I, P, L	HMG-CoA reductase inhibition, LDLR upregulation, PCSK9 suppression, and inhibition of cholesterol esterase and pancreatic lipase	[132,133,134]
Hypoglycemic	3–6	0 or +1	Mostly flexible/random-coil; short motifs favored	Hydrophobic residues located near Proline enhance DPP-IV inhibition	P, A, L, I, R, K, T, S, Y, M	Inhibition of α-amylase, α-glucosidase, DPP-IV; GLUT4 modulation; insulin-like activity	[162,163,187]
Antimicrobial	10–50	+2 to +11	α-helix, β-sheet (disulfide-stabilized), αβ, hairpinin	~50% hydrophobic residues	R, K, A, G, V, C	Disrupt microbial membranes	[204,226,228]
Antiviral	8–2′		Flexible/α-helical motifs	Amphipathic balance enhances viral interaction	C, P, G, I, D, N, V	Adsorption/receptor blocking, virucidal effects, and intracellular disruption of nucleic acid/protein synthesis	[199,229]

## Data Availability

The data presented in this study are available upon reasonable request from the author (S.A.-G.).
